# Facile synthesis of fluorescent Au/Ce nanoclusters for high-sensitive bioimaging

**DOI:** 10.1186/s12951-015-0071-y

**Published:** 2015-02-03

**Authors:** Wei Ge, Yuanyuan Zhang, Jing Ye, Donghua Chen, Fawad Ur Rehman, Qiwei Li, Yun Chen, Hui Jiang, Xuemei Wang

**Affiliations:** State Key Lab of Bioelectronics (Chien-Shiung Wu Lab), Department of Biological Science and Medical Engineering, Southeast University, Nanjing, 210096 China; School of Chemistry and Chemical Engineering, Southeast University, Nanjing, 211189 China

**Keywords:** Fluorescence bioimaging, Au/Ce nanoclusters, Probe, Tumor

## Abstract

**Background:**

Tumor-target fluorescence bioimaging is an important means of early diagnosis, metal nanoclusters have been used as an excellent fluorescent probe for marking tumor cells due to their targeted absorption. We have developed a new strategy for facile synthesis of Au/Ce nanoclusters (NCs) by doping trivalent cerium ion into seed crystal growth process of gold. Au/Ce NCs have bright fluorescence which could be used as fluorescent probe for bioimaging.

**Results:**

In this study, we synthesized fluorescent Au/Ce NCs through two-step hydrothermal reaction. The concentration range of 25–350 μM, Au/Ce NCs have no obvious cell cytotoxicity effect on HeLa, HepG2 and L02 cells. Furthermore, normal cells (L02) have no obvious absorption of Au/Ce NCs. Characterization of synthesized Au/Ce NCs was done by using TEM, EDS and XPS. Then these prepared Au/Ce NCs were applied for *in vitro/in vivo* tumor-target bioimaging due to its prolonged fluorescence lifetime and bright luminescence properties.

**Conclusions:**

The glutathione stabilized Au/Ce NCs synthesized through hydrothermal reaction possess stable and bright fluorescence that can be readily utilized for high sensitive fluorescence probe. Our results suggest that Au/Ce NCs are useful candidate for *in vitro/in vivo* tumor bioimaging in potential clinical application.

## Background

Cancer is still a serious threat to human health and its effective treatment is still a big challenge. Its early diagnosis provides opportunity for the effective treatment and hence can improve the survival rate. The early diagnosis have been researched extensively through finding biomarkers [1,2] and *in situ* fluorescent bio-imaging [3,4]. Fluorescence imaging has been introduced as an important bioimaging tool and *in vivo* fluorescence imaging can improve the visibility of infected site.

Recently, many fluorescent composites have been developed as sensitive optical imaging probes, including fluorescent dyes [5,6], quantum dots [7-10], metal nanoparticles [11-13] and up-converted nanomaterials [14,15]. Precious metals like gold and silver nanomaterials have been paid much attention for application in a wide range of biomedical field due to their excellent biocompatibility and physicochemical properties [16-18]. Folic acid conjugated AuNCs@SiO_2_ nanoprobes (AuNCs@SiO_2_-FA) with good biocompatibility have been designed and applied into fluorescent imaging of gastric cancer cells [19]. Meanwhile, various kinds of metal nanoclusters have been synthesized by coating biological ligands such as BSA, PEG, DHLA and GSH, etc., and accordingly, different kinds of biomedical function have been developed to meet the practical and clinical needs [17]. In addition, lanthanide-doped luminescent nanomaterials have been also explored for some disease diagnosis [20,21]. Ce^3+^ and Eu^3+^ co-doped multifunctional nanoparticles like NaGdF_4_:Ce^3+^, Eu^3+^ NPs have been also explored for bioimaging [21]. Moreover, some studies reported the combining of various detection mediums like quantum dots and magnetic nanoparticles for multi-mode imaging [22]. In view these observations, a new strategy for facile synthesis of fluorescent Au/Ce nanoclusters (NCs) has been explored in this contribution by doping trivalent cerium ion into seed crystal growth process of gold. Through hydrothermal synthesis of these glutathione stabilized nanoclusters, it is possible to utilize the biocompatible and fluorescent Au/Ce NCs to realize high-sensitive *in vitro/in vivo* tumor-target bioimaging.

## Results and discussion

### Synthetic strategy

**Figure 1:**
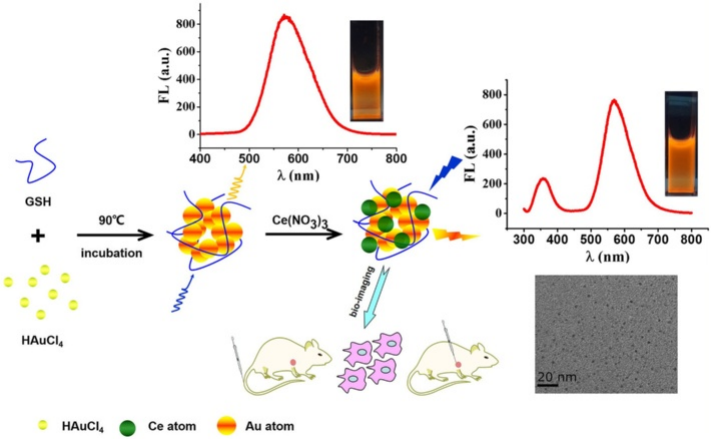
**Illustration of the synthesis of fluorescent GSH–Au/Ce NCs.** Mixed with a certain concentration of glutathione and HAuCl_4_ until the solution became colorless, then the mixture was placed in a water bath with 90°C for two hours, and a certain concentration of Ce(NO_3_)_3_ aqueous solution was added immediately, the mixture was blending and heating in water bath with 90°C for another one hour. The separated and purified Au/Ce NCs were applied in bioimaging and measured by fluorescence spectrometer and transmission electron microscope.

Considering the multiple roles played by glutathione (GSH) in cell survival and metabolic functions, GSH functionalized nanocomposites were prepared and tested. This study represents promising tools for a wide variety of investigations in biomedical field. As shown in Figure 1, the facile synthesis of GSH protected Au/Ce NCs could be readily realized to obtain well dispersible Au/Ce nanoclusters. It is noted that after mixing a certain concentration of GSH and HAuCl_4_, one step hydrothermal reaction assisted the synthesis of Au nanoclusters, where GSH acted as a reductant and stabilizing agent. Afterwards, a certain amount of CeNO_3_ was added to the as-synthesized solution that facilitated the realization of stable ultra-small Au/Ce nanoclusters. The end-products of fluorescent Au/Ce nanoclusters were well separated and purified by ethanol centrifugal.

### Characterization of Au/Ce NCs and Au NCs

**Figure 2:**
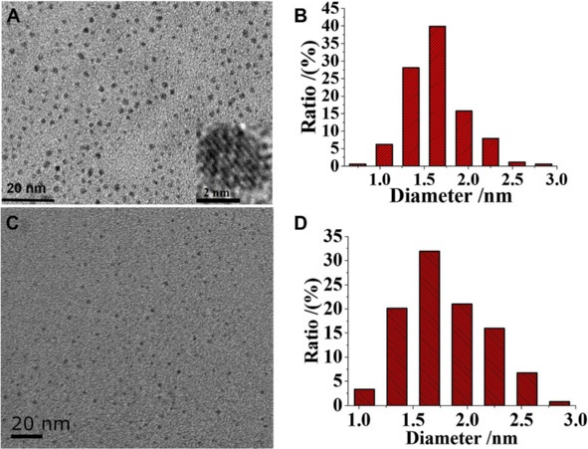
**Transmission Electron Microscope (TEM) images. (A)** Typical image of Au NCs. **(C)** Typical image of Au/Ce NCs. Inset in image **(A)**: high resolution image with the crystallinity of the metallic structure. **(B)** The size distribution histogram of Au NCs. **(D)** The size distribution histogram of Au/Ce NCs.

Transmission Electron Microscopy (TEM) images of GSH capped Au/Ce NCs illustrated the perfect dispersion of the nanocomposites without any aggregation. Figure 2 showed typical images of purified Au NCs and Au/Ce NCs that can disperse well in ultrapure water. This typical TEM image of the resulting Au NCs and Au/Ce NCs evidenced their high mono-dispersion and relatively uniform sizes. As shown in Figure 2, TEM characterization demonstrates that 90% of the Au NCs (Figure 2A) and Au/Ce NCs (Figure 2C) ranged between 1.2–2.2 nm in diameter (i.e., with narrow size distribution), while the high resolution image (HRTEM) of Au NCs showed clear crystal of metallic structure. HRTEM (Figure 2A, inset) illustrated that the gold nanoclusters kept their interplanar Au–Au spacing at ca. 0.2 nm.

**Figure 3:**
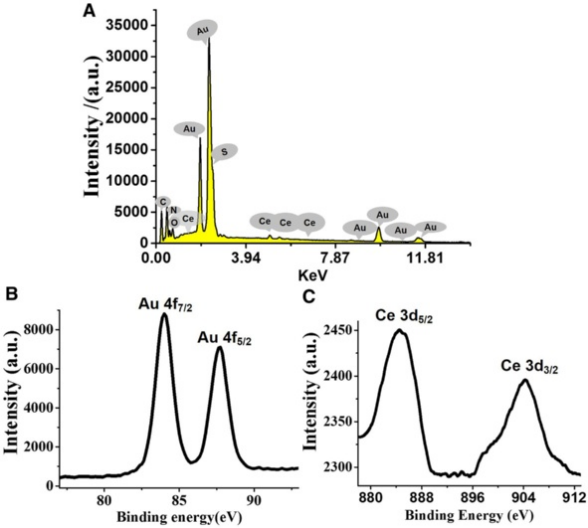
**Elemental analysis of Au/Ce NCs. (A)** EDS of the Au/Ce NCs formed by hydrothermal synthesis. **(B)** and **(C)** were X-ray photoelectron spectra (XPS) evidencing the Au 4f and Ce 3d photoelectron emission from the Au/Ce NCs, respectively.

Moreover, X-ray photoelectron spectroscopy (XPS) and energy dispersive X-ray spectroscopy (EDS) were used to investigate the valence of gold and cerium in the Au/Ce NCs after the formation of relevant nanoclusters. The EDS analysis indicated that Au and Ce elements co-exist in the composites without other elemental impurity present in the prepared Au/Ce NCs (Figure 3A). As shown in Figure 3B, two peaks located at the binding energy of 83.9 and 87.7 eV were observed, which were consistent with the emission of 3d photoelectrons from Au (0), while cerium’s 3d orbital spectrum with prominent Ce^3+^ peaks at 884.8 and 904.2 eV, respectively, as earlier reported [23].

**Figure 4:**
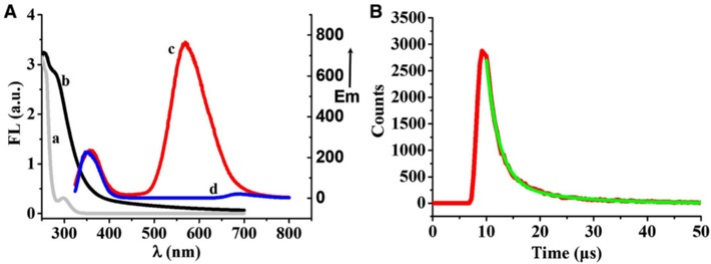
**Optical characterization of Au/Ce NCs. (A)** UV–Vis absorption spectroscopy and fluorescence emission spectrum: Curve a and curve b were UV–Vis absorption spectroscopy of Ce^3+^ aqueous solution and Au/Ce NCs, respectively. Curve c and curve d were fluorescence emission spectrum of Au/Ce NCs and Ce^3+^ aqueous solution, respectively, excitation wavelength was 290 nm. **(B)** Fluorescence lifetime analysis of Au/Ce NCs, excitation was 430 nm.

Fluorescence and UV–Vis absorption spectroscopy were further utilized to characterize the optical properties of the GSH capped Au/Ce NCs. As shown in Figure 4A, fluorescence emission peak of Ce^3+^ solution appeared at 350 nm, while two apparent emission bands of Au/Ce NCs characteristic peak of gold and cerium located at ca. 570 nm and 360 nm, respectively. The peak of trivalent cerium red shifted for about 10 nm, which may attributed to the forming of Au/Ce NCs. In addition, curve a showed UV–Vis absorption peak of Ce^3+^ aqueous solution; it is evident that UV–Vis absorption peak appeared at ca. 290 nm, while the relevant absorption peak of Au/Ce NCs was almost smeared out due to the formation of the hybrid Au/Ce NCs, thereby suggesting the successful formation of Au/Ce nanoclusters. Based on these observations, we believe that Ce^3+^ ions doped in the lattice of gold during seed crystal growth process affected the optical properties of Au/Ce NCs.

Several noble metal clusters have shown remarkably long fluorescence lifetimes of 1 or 2 orders of magnitude higher than organic dyes and quantum dots [24]. As shown in Figure 4B, the fluorescence lifetime determined by time-correlated single photon counting indicates that Au/Ce NCs exhibit a luminescence lifetime with a biexponential decay; a short component τ_1_ (2.34 ± 0.05 μs, 87.2%) and a long component τ_2_ (11.25 ± 0.30 μs, 12.8%), respectively. The microsecond time scale long lifetime component maybe due to the electron transfer from the clusters to the ligand where a redox process might occur and charge-separated trap [25], hence, making it possible to avoid the interference of short-lived background fluorescence. The apparent long fluorescence lifetime of Au/Ce NCs makes it possible for their future bio-application in monitoring some important biological process through biosensing or bioimaging.

### Application of Au/Ce NCs in bioimaging

**Figure 5:**
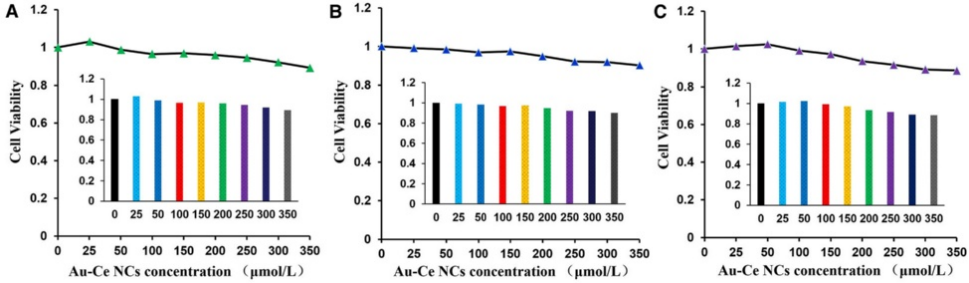
**Measurement of cell viability.** MTT assay assessment of dose-dependent cytotoxicity towards HepG2 cells **(A)**, HeLa cells **(B)** and L02 cells **(C)** after incubation with Au/Ce NCs solutions for 24 h.

Based on the above observations, we thus examined the cytotoxic activity of Au/Ce NCs against cancer cell lines (HeLa and HepG2 cells) and normal cells (L02) using MTT assays. MTT assay revealed that Au/Ce NCs have good compatibility even at relatively high concentrations (Figure 5).

**Figure 6:**
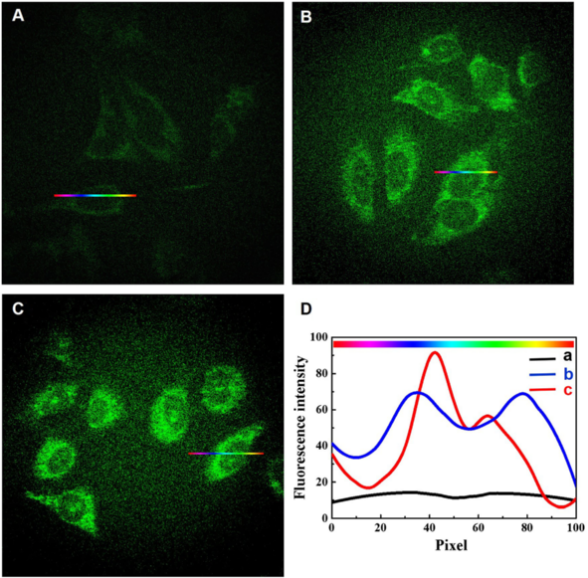
**Laser confocal fluorescence micrographs of HeLa cancer cells.** HeLa cells incubated in the absence of Au/Ce NCs **(A)**, in the presence of 50 μmol/L **(B)** and 150 μmol/L **(C)** Au/Ce NCs solutions for 24 h. **(D)** Relative fluorescence intensity variations along cross-sections a (in A), b (in B), or c (in C) (the color gradient coding illustrates the direction of the sampling). Fluorescence micrographs were collected by using a 488 nm fluorescence excitation wavelength.

**Figure 7:**
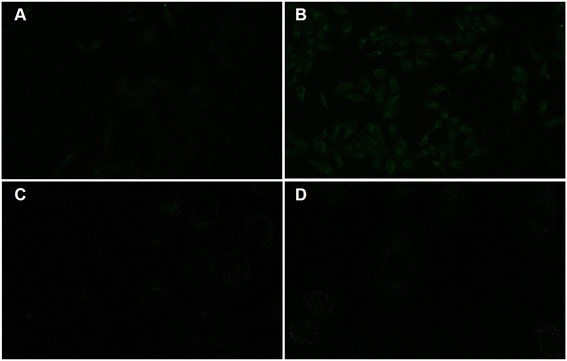
**Laser confocal fluorescence micrographs of HepG2 cells and L02 cells. (A)** HepG2 cells without Au/Ce NCs treatment. **(B)** HepG2 cells treated with 150 μmol/L Au/Ce NCs solutions for 24 h. **(C)** L02 cells without Au/Ce NCs treatment. **(D)** L02 cells treated with 150 μmol/L Au/Ce NCs solutions for 24 h. The micrographs of HepG2 cells were acquired by 20× IR coated objective. The micrographs of L02 cells were collected by 63× IR coated objective. The excitation wavelength was 488 nm.

The bright green cellular fluorescence of Au/Ce NCs inside HeLa cancer cells appeared to be adequate for use in the *in vivo* bio-imaging of relevant live tumor cells. It is evidenced in Figure 6 that the Au/Ce NCs were well distributed in the cells so that the relevant edges and morphologies of the cells were neatly delineated. Importantly, the fluorescence intensity increased with increase of incubation time and concentration of Au/Ce NCs (Figure 6A–C). The relative fluorescence intensity variations are further confirmed by a comparison of the quantitative variations in the fluorescence intensities across both cell types, as shown in Figure 6D. Similarly, we also explored that HepG2 and L02 cells support the bio-labeling of Au/Ce NCs. Figure 7 showed that HepG2 treated with Au/Ce NCs displayed clear fluorescence. In contrast, little or almost no intracellular fluorescence was observed in control group involving L02 cells, which showed that there was no obvious fluorescence for normal cells subjected to the same incubation conditions (i.e., in the presence of Au/Ce NCs) as provided for the HeLa cancer cells. Therefore, Au/Ce NCs can be readily applied into *in vitro* / *in vivo* bio-imaging of relevant tumor cells.

**Figure 8:**
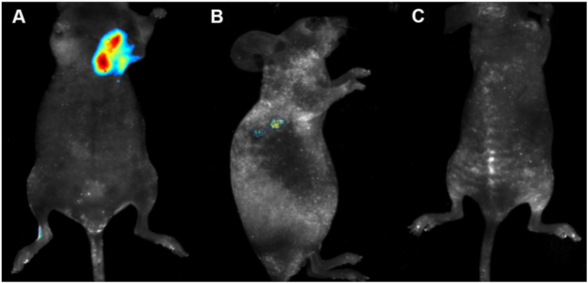
**Representative xenograft tumor nude mice models of Cervical carcinoma** ***in vivo*** **imaging. (A)** *In vivo* fluorescence imaging 24 h after a subcutaneous injection of 5 mmol/L Au/Ce NCs solution near the tumor. **(B)** *In vivo* fluorescence imaging 24 h after a intravenous injection 5 mmol/L Au/Ce NCs solution through the tail. **(C)** Control nude mice without tumor after a intravenous injection equivalent PBS through the tail. Fluorescent Au/Ce NCs were observed inside the tumors using a 455 nm excitation wavelength.

On the basis of aforementioned *in vitro* results, we now wish to establish the feasibility of *in vivo* bio-imaging of tumors by fluorescence based on fluorescent Au/Ce NCs. For this purpose, we relied on a xenograft tumor model of Cervical carcinoma. As shown in Figure 8, subcutaneous injection of Au/Ce NCs around xenograft tumors allowed the clear observation of bright fluorescence around the tumor after 24 hours while the fluorescence in the mouse injected with intravenous injection Au/Ce NCs solution through the tail was also observed by *in vivo* fluorescence. No obvious toxic effects were observed during the experimental trail, suggesting that Au/Ce NCs solutions can be administered for high-sensitive *in vivo* tumor-targeted bioimaging.

## Conclusions

In summary, a novel strategy of facile Au/Ce nanoclusters synthesis by doping trivalent cerium ion into seed crystal growth process of gold has been developed. The EDS, UV–Vis absorption spectroscopy, fluorescence and XPS characterization validates that the glutathione stabilized Au/Ce NCs via hydrothermal synthesis possess stable and bright fluorescence. The as-prepared GSH–Au/Ce NCs have strong fluorescence and long fluorescence lifetime, which could be readily utilized for high sensitive tumor-target bioimaging.

## Methods

### Chemicals, materials and cells

Auric chloride acid (HAuCl_4_ · 6H_2_O), L-glutathione (GSH) and Cerium nitrate (Ce(NO_3_)_3_ · 6H_2_O) along with all chemicals (analytical reagent, AR) were purchased from Sinopharm Chemical Reagent Co., Ltd. DMEM medium, fetal bovine serumwere (FBS) and penicillin were purchased from SunShineBio Technology Co., Ltd (Nanjing, China). Ultrapure water (18.2MU cm; Milli-Q, Millipore) was used as lyticagent of all aqueous solutions reagent. Tumor cells like HeLa cells, HepG2 cells and normal cells like L02 cells (purchased from Cell Bank of Chinese Academy of Sciences, Shanghai) were applied in our study. All of them were cultured in DMEM medium supplemented with 10% fetal bovine serum and 1% penicillin at 37°C in a carbon dioxide cell incubator with 5% CO_2_ and 95% relative humidity.

### Characterizations

Transmission Electron Microscopy (TEM) images were recorded using a JEM-2100 microscope (JEOS, Japan) to characterize the size and size distribution, a certain concentration of sample solution was spotted on carbon coated copper grid (300 meshes) and was dried in desiccator at room temperature. Energy dispersive X-ray spectroscopy (EDS) analyses was done in a Zeiss Ultra Plus scanning electron microscopic (SEM). The valence state of gold and cerium atoms in the Au/Ce nanoclusters was investigated by a PHI 5000 VersaProbe X-ray photoelectron spectrometer (XPS), briefly, samples were droped on a silicon wafer and dried in laboratory ambience to form evenly spread film. Thermo BioMate 3S UV–visible spectrophotometer was used for the UV–Vis absorption measurements, spectra were typically measured in the range of 200–700 nm. Photoluminescence spectra were carried out using SHIMADZU RF-5301 PC instrument. Cells fluorescence imaging were collected by laser scanning confocal microscope Carl Zeiss LSM710 (Zeiss, Germany). Nude mice *in vivo* imaging were carried on vivo multispectral imaging system (Maestro EX).

### Preparation of glutathione stabilized Au nanoclusters and Au/Ce nanoclusters

Precisly prepared 2.4 mM glutathione aqueous solution, measured out 8.5 μL pre-prepared 1 M HAucl_4_ aqueous solution and added into 5 mL 2.4 mM GSH solution, and shaked vigorously until the solution becomes colorless. The final concentration of HAuCl_4_ was 1.7 mM. Then the mixture was placed in a water bath with 90°C for two hours to obtain low fluorescence Au seed crystal and added 5 μL 1 M Ce(NO_3_)_3_ aqueous solution immediately. The mixture was blending and heating in water bath with 90°C for another one hour. A illustration need to be added that the synthesis of pure Au nanoclusters were performed in same condition without adding cerium ion. The products were separed and purified by centrifugation at 10000 rpm for 10 min with 1:4 ethanol. The precipitation were redispersed in deionised water or phosphate buffer solution (PBS, pH 7.2) for further application.

### Cell growth inhibition study by MTT assay

Briefly, 100 μL medium with 2 × 10^3^ Cells/well were plated in 96-well plates, after about eight hours incubation, cells were treated with 100 μL various concentrations of Au/Ce NCs medium solution. Each concentration set up five repetition. After treatment for 24 hours, 20 μL MTT solution (5 mg/ml) was added to each well. And cells were incubated for another four hours, then the supernatant was removed and 150 μL DMSO was added per well. Samples were then shaked well for 10 min and the optical density (OD) was read at a wavelength of 490 nm by microplate reader (MK3, ThermoFisher). All experiments were performed in triplicate.

### Construction of the xenografted tumor mouse model

BALB/c female athymic nude mice, age-matched (four weeks of age) and weight-matched (18–22 g), were purchased from Peking University Health Science Center. All experiments involving mice were approved by the National Institute of Biological Science and Animal Care Research Advisory Committee of Southeast University, and experiments were conducted following the guidelines of the Animal Research Ethics Board of Southeast University. The mice were randomly assigned to groups for experimental purposes. They were maintained in clean facilities with a 12-hour light/dark cycle and received water and food through a semi-barrier system. Subcutaneous tumor models were generated by the subcutaneous inoculation (0.10 mL volume containing 5 × 10^7^ cells/mL media) of HeLa cells in the right side of their armpit or nearby sites using a 1-mL syringe with a 25 G needle. Tumor growth was monitored until a palpable size for next applications.

### *In vitro* and *in vivo* bioimaging study

For cellular imaging, HeLa cells and L02 were treated with a certain concentrations of Au/Ce NCs solutions and incubated at 37°C for 24 h. The cells were washed three times with PBS before fluorescence imaging. A 488-nm excitation laser beam (Andor Revolution XD) was focused using a 63× IR coated objective (Nikon). Similarly, HepG2 cells were treated with 150 μmol/L Au/Ce NCs solutions, the cells were washed three times with PBS before fluorescence imaging. A 488-nm excitation laser beam (Andor Revolution XD) was focused using a 20× IR coated objective (Nikon).

For *in vivo* bio-imaging of Au/Ce NCs in the tumor location complex solution was administered into the solid tumor mouse model through local injection or intravenous injection through the tail. The mice were fully anesthetized by gaseous 5% isoflurane anesthesia. The *in vivo* bio-images were acquired on Cri Maestro *in vivo* imaging system. After incubation for 24 h, fluorescent Au/Ce nanoclusters were observed inside the tumors by *in vivo* fluorescence imaging using a 455 nm excitation wavelength. In comparison, the negative control groups, which received an equivalent volume of Phosphate Buffered Saline (PBS), did not exhibit any apparent fluorescence. The ROI (regions of interest) analysis was measured under the assistance of CRi Maestro Image software. The studies were approved by the National Institute of Biological Science and Animal Care Research Advisory Committee of Southeast University, while experiments conducted the guidelines of the Animal Research Ethics Board of Southeast University.
